# A high-resolution wind damage model for Europe

**DOI:** 10.1038/s41598-020-63580-w

**Published:** 2020-04-22

**Authors:** E. E. Koks, T. Haer.

**Affiliations:** 1 Institute for Environmental Studies, Vrije Universiteit Amsterdam, Amsterdam, The Netherlands; 2 Environmental Change Institute, University of Oxford, Oxford, United Kingdom

**Keywords:** Environmental impact, Natural hazards

## Abstract

Extreme wind events are among the costliest natural disasters in Europe, causing severe damages every year. Despite the significant impact, damages related to windstorms are an understudied topic in academia. For damage estimates, the community mostly relies on post-disaster insurance data, which is often not publicly available. Few studies offer more generic tools, but again these are often based on non-disclosed insurance data. To offer a generic, high-resolution, reproducible, and publicly accessible tool, this study presents a wind damage model that is built around publicly available hazard, exposure, and vulnerability data. We apply the model to assess building damages related to extratropical storms in Europe, but the methodology is applicable globally, given data availability, and to other hazards for which similar risk frameworks can be applied. The results show that for Europe, coastal regions are affected the most, with the United Kingdom, Ireland, Germany, France, the Netherlands, and Denmark as most affected countries. We find that the modelled damage estimates are in line with reported damages for a series of historical storms. The model is distributed as an open-source model to offer a transparent and useable windstorm damage model to a broad audience.

## Introduction

Extreme weather events cause increasing havoc throughout the world. Between 2012 and 2017, extreme weather events accounted for over 55% of the overall global damage and 72% of the insured damage caused by natural disasters^1^. Recent studies have shown that the increased occurrence of these events, such as wind and hail storms, can be (causally) linked to climate change^2,3^. For example, the severity of storm Desmond in 2015^4^ and the extreme precipitation event of 2014 in the Netherlands^5^ have both been partly attributed to a changing climate^6^. In the future, it is expected that the frequency and intensity of extreme weather events may increase further due to anthropogenic influences^7,8^.

In Europe, examples of damaging storms are numerous and occur on an almost yearly basis. In 1999, storm Lothar caused severe damages across France with total recorded damages of up to 8 billion USD^9^. More recently, in 2010, Storm Xynthia caused total damages of over 6 billion USD in large parts of western Europe^1^. Not surprisingly, due to the large damages to (insured) assets, the impacts of windstorms are extensively recorded and modelled by the insurance industry. Most European academic studies in the field of disaster risk modelling, however, tend to focus on flood and earthquake damage assessments^10,11^. The few windstorm damage studies that are conducted have focused primarily on Central Europe^12,13^ or are quick damage assessments by the European Severe Weather Laboratory^14^. To our knowledge, only one study has assessed the European-wide consequences of insured wind storm damages^15^, but no models have been developed yet to assess damages on a building level. Moreover, most studies, such as Schwierz *et al*.^15^, use insurance data which is often not publicly accessible, limiting reproducibility and usability. To fill these gaps, this study presents a high-resolution building-level windstorm damage model for the whole of Europe, developed with open-source data, and distributed as open source.

The model developed in this study evolves from building footprint data extracted from OpenStreetMap (OSM). In recent years, OSM has become ever more complete and is reliable enough to be used for risk assessment tools^16^. To exemplify the use of the damage model, we estimate the damages for 53 historical windstorms for 21 countries in Europe. These countries are selected based on reported historic damages^9^. Those countries that show negligible damages for extratropical storms are not considered in this analysis^9^. Wind damages are estimated by combining historical storm footprints with building type specific stage-damage curves. As this is one of the first studies to perform such an analysis on a continent scale, damages are validated using historical data and model parameters are tested through a global sensitivity analysis.

## Results

### Single storm damage estimates

**Figure 1:**
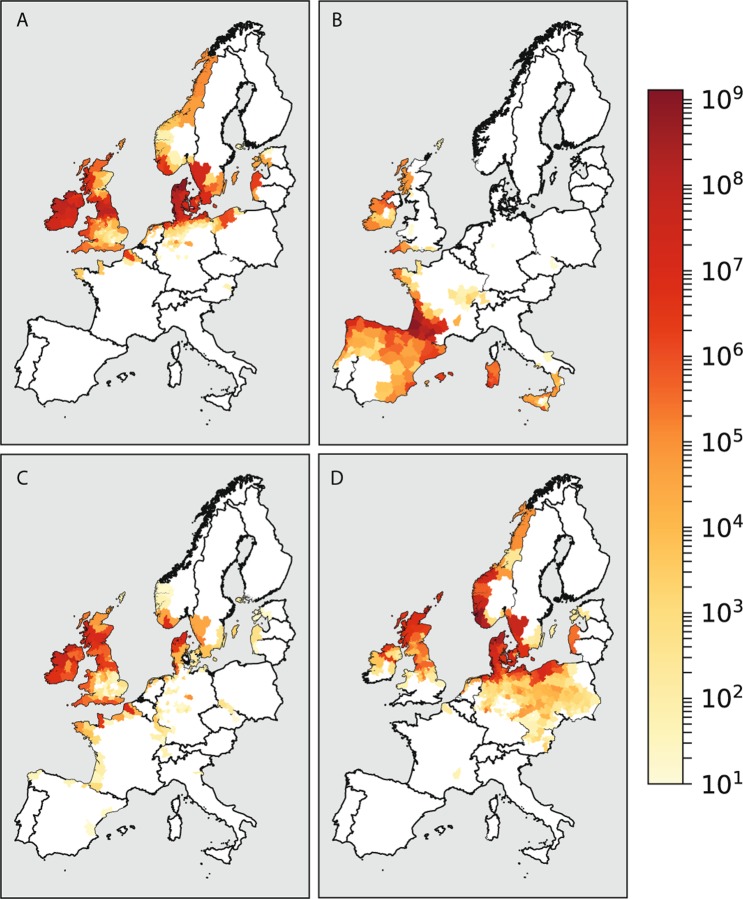
Damage estimates (USD) for **(A)** storm Erwin, **(B)** storm Klaus, **(C)** storm Ulli, and **(D)** storm Xaver.

Figure 1 shows four examples of damage estimates aggregated for each Nomenclature of Territorial Units for Statistics (NUTS) 3 region in Europe. The storms are selected based on their distinctive different tracks, and the results are shown on a logarithmic scale to emphasize spatial differences. All results are presented as absolute values for each NUTS3 region, as prioritization of adaptation or insurance policies is commonly based on aggregate damages rather than damages normalized for spatial extent or building count of the NUTS3 region. Storm Erwin (Fig. 1A, also named Gudrun) developed as a low-pressure system, strengthening while moving over the north Atlantic towards the coast of Northern Ireland and Scotland in 2005. Major damages occurred in Cumbria in the United Kingdom. After passing over the United Kingdom, it moved towards Denmark and Sweden before passing into the Baltic Sea. Erwin caused major damages in the south of Sweden, and throughout Denmark with the northern regions affected the most^17^. Storm Klaus (Fig. 1B) was a windstorm that made landfall over (large) areas of Spain, central and southern France, and Italy in 2019. Reported peak gust were over 200 km/hour, with sustained winds of 170 km/hour. The storm caused major damages in the south of France and throughout Spain. Storm Ulli (Fig. 1C) developed of the coast of the United States, moving rapidly across the Atlantic, passing over Ireland, Northern Ireland, England, and Scotland in 2012. It travelled over the North Sea towards Denmark where it caused major damages, eventually reaching Sweden. Storm Xaver (Fig. 1D) developed off the coast of Iceland and affected large parts of Northern Europe in the winter of 2013. It moved mostly over southern Norway, Denmark, and southern Sweden, after which it affected large parts of Poland before dissipating.

The modelled spatial distribution of the affected regions for each of the four storms are in line with the reported storm impacts in the Extreme Wind Storms (XWS) catalogue^9^, and several reports on the specific storms^17–19^. The XWS catalogue is a database of storm tracks, model-generated maximum 3-second gust footprints and provides estimates of insured damages for each wind storm included in the database. Not only are the overall geospatial patterns in line with reported damages, but also the location of the most affected areas. For storm Erwin, we find the highest damages in Cumbria (UK) and Denmark^17^, southern France and northern Spain for storm Klaus^18^, northern Denmark for storm Ulli^9^, and southern Scandinavia, Denmark, and northern Poland for storm Xaver^19^.

The modelled damages for storm Erwin are 2.6 billion USD, for storm Klaus are 2.7 billion USD, for storm Ulli 0.2 billion USD, and for storm Xaver 1.5 billion USD. The XWS catalogue reports 2.2, 3.5, 0.2, and 0.9 billion USD for the four storms, respectively. When we take into account that the XWS catalogue reports only insured damages, and that our wind damage model calculates all damages, the results are in line with reported values, giving us increased confidence in the methodology. **Section 2.4** provides more validation of our results.

### Total damage and risk estimates

**Figure 2:**
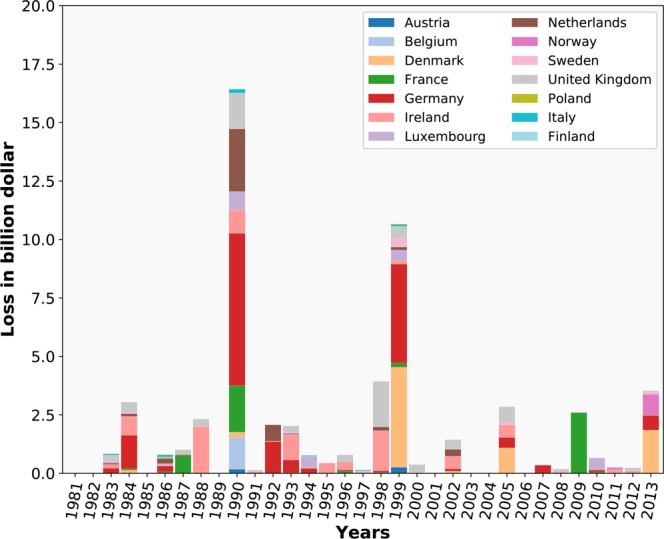
Historical damages per country per year.

Figure 2 provides an overview of the total historical damages for the most damaged countries in each year, as estimated through our damage model using the most recent exposure portfolio from OSM. The damages are estimated in dollar damage values of the year 2012 and as if the storm were to occur in the present day, similar to the XWS catalogue^9^ and comparable to the approach by Waisman (2015), as presented in Table S5. As becomes apparent from Fig. 2, Germany is, in absolute terms, the most vulnerable for extratropical storms, having a large share of the total damage for almost every year. Especially in the years 1990 and 1999, when several big storms passed over central Europe and Germany. Those years also stand out as the most damaging between 1981 and 2013. In 1990, several big storms hit Europe, such as Herta, Wiebke, Vivan, and Daria. Daria alone caused reported insured damages of 8.2 billion USD, primarily in Belgium, France, Germany, the Netherlands, and the United Kingdom. In 1999, Europe was hit by Anatol, Martin and Lothar, where the latter caused reported insured damages of 8 billion USD. Fig. S4 presents the same damages as Fig. 2, but per sector. The results show that damage to buildings in residential areas (as indicated by the Corine Land Cover (CLC) classification) are dominating the total damages in each storm. Impacts on transport related buildings are relatively minor and damage to agricultural related buildings and industrial/commercial buildings are somewhat similar.

**Figure 3:**
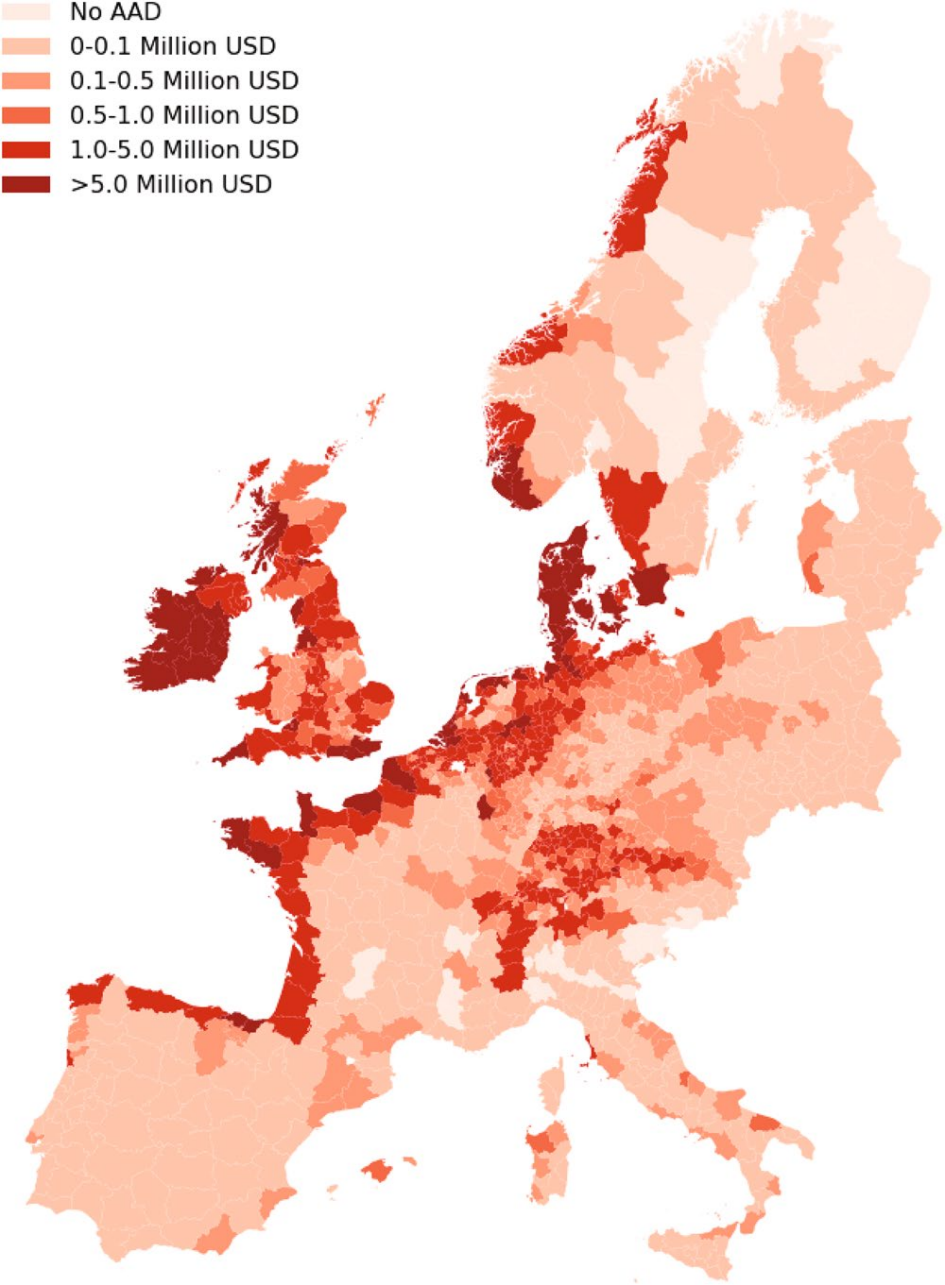
Total average annual damages (AAD) per NUTS3 area in Europe.

Figure 3 presents the historical average annual damages (AAD) over the forty years of storm data that is used in this study. The results show that coastal regions are most at risk to wind storm damage. This is not surprising, as most violent storms are generated over open sea, losing wind speed when making landfall. The British Isles face heightened risk throughout the countries, as they lay in the path of many extratropical storms moving from west to east. Other countries are mostly affected within their coastal areas, except for Denmark and countries surrounding the Baltic Sea, which face high risk also further inland. A few surprising results can be seen as well, such as the areas at risk in Italy and the eastern part of Spain. The same is the case for inland Poland and parts of the Czech Republic. However, major storms do tend to affect the central European countries, such as storm Kyrill in 2007 and storm Xaver in 2013 (see Fig. 1D).

### Sensitivity and uncertainty analysis

To get a sense of the model’s performance, we test the influence of choosing different fragility curves and alternative assumptions concerning building use (see Methods). In this study, a fragility curve is defined as the relation between the intensity of the storm (the x-axis) and its relative impact to the building asset (the y-axis). The results of the uncertainty analysis show that the outcome of the windstorm damage model can vary substantially, with certain parameter settings resulting in substantially higher damages compared to other settings. This distribution indicates that one cannot simply assume a certain set of parameter settings, without validating the outcomes. The results show that for the smaller countries the mean of the outcomes is more comparable to the vendor model estimates^20^ than the mean of the outcomes for the larger countries. The larger countries show more quickly an overestimation of the damage, indicating that (i) a different set of fragility curves should be tested for these countries and (ii) there is a large regional differentiation in building type and value, meaning that a one-size-fits-all curve will be difficult to implement for large countries.

**Figure 4:**
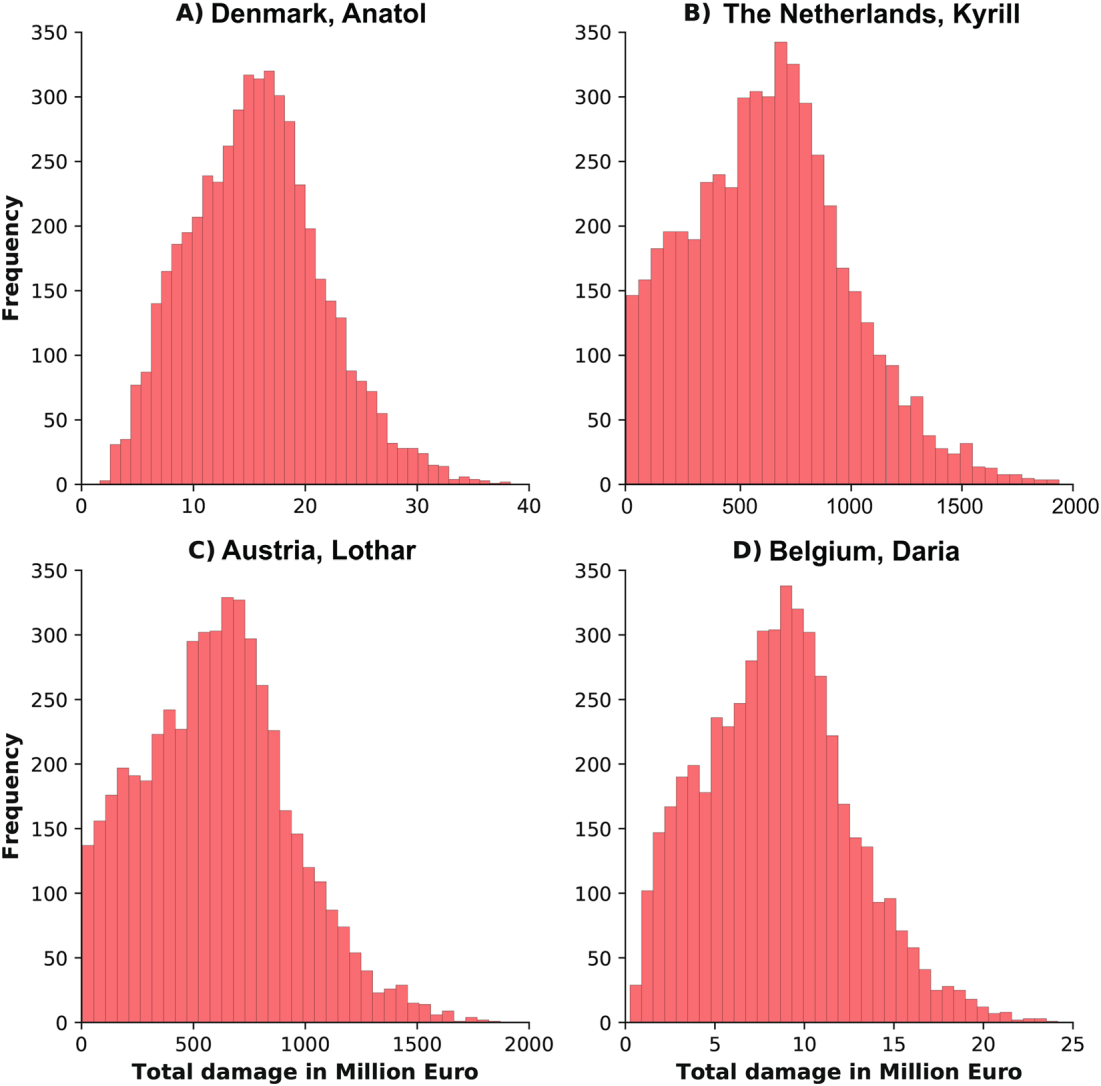
Four examples of the range in model outcomes as calculated with the damage model. Panel (A) shows the frequency of damages for storm Anatol in Denmark, panel (B) for storm Kyrill in The Netherlands, panel (C) for storm Lothar in Austria and panel (D) for storm Daria in Belgium.

Figure 4 shows histograms for Denmark, The Netherlands, Austria and Belgium, illustrating the range of damages estimated using the parameter set. All cases show a skew towards the right, indicating that a specific set of parameter values (i.e. steep fragility curve in an urban area, see Methods) may result in substantially higher damages compared to the mean. The results show a large variation in model outputs both within each case, and between cases. The upper left panel in Fig. 4, for instance, showing the outcomes of storm Anatol for Denmark, has estimates ranging from almost 0 up to 35 billion Euros of damage. The average total damage for Anatol, estimated by the four vendor models, however, is only around 2.5 billion Euro, which is at the lower end of the range estimated in this analysis. When comparing the outcomes of Fig. 4 with the vendor model estimates in Table S5, there seems to be a higher tendency to overestimate for the larger countries, than for the smaller countries, such as Ireland and Luxemburg. For the smaller countries, the damages calculated through the damage model show an average that is very similar to the average estimated damages of the four vendor models^20^. This tendency can be explained by two reasons. Firstly, it indicates that there is a large regional differentiation in building types and reconstruction costs (value of housing) in the larger countries. This makes it difficult to find a correct ‘average’ curve to use for all the regions in a country. Secondly, it may indicate that for some countries, such as the larger countries, a fragility curve should be used which is less steep or starts at higher wind speeds.

**Figure 5:**
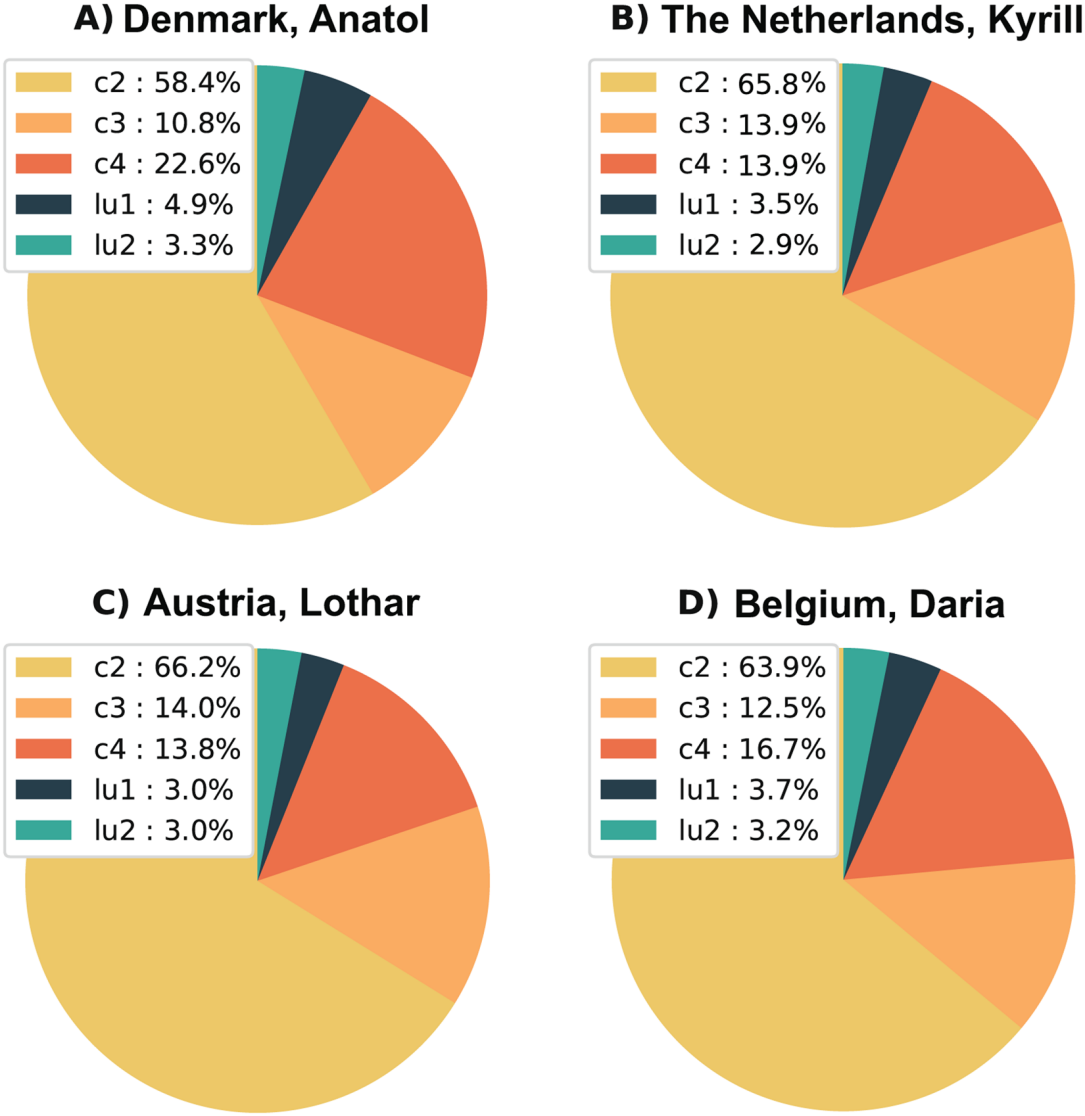
Outcomes of the sensitivity analysis for four storm/country combinations. Panel (A) shows the outcome of the sensitivity analysis for storm Anatol in Denmark, panel (B) for storm Kyrill in The Netherlands, panel (C) for storm Lothar in Austria and panel (D) for storm Daria in Belgium. Three fragility curves are represented by ‘c2’, ‘c3’ and ‘c4’. The ratio of residential & commercial to industrial in urban is represented by ‘lu1’ and in rural by ‘lu2’.

Figure 5 shows circle diagrams for four combinations of country/storms, illustrating the relative influence of each of the parameters considered in the sensitivity analysis on the damage estimations. The higher the share of a specific parameter, the more it influences the damage outcomes when it changes to a different setting. As becomes apparent from Fig. 5, the steepest fragility curve considered (c2) has the highest influence on the damage modelling outcomes, following by c3 and c4, respectively. The ratio of residential & commercial to industrial in urban (lu1) and rural (lu2) have the least influence in all cases considered in this study. These results are not unexpected. The fragility curves determine the damage ratio for specific gust speeds. The steeper the curve, the higher the damage ratios are at lower gust speeds. A high share of curve ‘c2’ will result in high damages, whereas a low share of curve ‘c2’ will result in low damages.

### Validation of results

Estimating each of the historical damages in line with other models, or as the observed damage from Swiss Re reported in the XWS catalogue^9^, has proven to be difficult. This, however, should not come as a surprise. Even within the different vendor models there is a wide range in the damage estimations (in some cases a factor four difference between their estimates). This indicates that all models behave differently, use very different vulnerability curves (perhaps even different curves for different storms) and, most likely, each of the models has been calibrated on different portfolios. This makes it difficult to validate our methodology to the vendor models, as it is unclear which of these performs better.

When comparing our results to the XWS catalogue (Table 1), it becomes apparent that most storms show lower damage estimates compared to the XWS catalogue damages^9^. Others are in the same range, such as Daria, Herta, Vivian and Christian. Interestingly, most of these are 1990 storms. Especially the storms between 2007 and 2010 show much lower estimates. This may indicate that several storms in this time-period, among which Kyrill, were storms with local convective behavior. Local convective behavior is often not properly captured in the hazard data due to the resolution of the input data, and is something which can be addressed when higher resolution windstorm footprints become available.

**Table 1:** Comparison of estimated damages with Swiss Re observed insured damages reported in the XWS catalogue.

Storm name	Date	Observed Insured damage (USD, indexed to 2012)	Damage estimated through damage model	Damage estimated through damage model (corrected for OSM coverage ratio in Table S2)
Great Storm of 87	16-10-1987	6.3bn	1.9bn	3.3bn
Daria (Burns’ Day storm)	25-1-1990	8.2bn	8.1bn	13.5bn
Herta	3-2-1990	1.5bn	1.6b	2.2bn
Vivian	26-2-1990	5.6bn	5.6b	10.5bn
Wiebke	28-2-1990	1.4bn	0.6bn	1.7bn
Anatol	3-12-1999	2.6bn	6bn	9.9bn
Lothar	26-12-1999	8.0bn	2.8bn	5.6bn
Martin	27-12-1999	3.3bn	2.3bn	4bn
Erwin (Gudrun)	8-1-2005	2.2bn	2.6bn	6.6bn
Gero	11-1-2005	0.6bn	0.3bn	1bn
Kyrill	18-1-2007	6.7bn	0.4bn	0.6bn
Klaus	24-1-2009	3.5bn	2.7bn	5.6bn
Xynthia	27-2-2010	2.9bn	0.7bn	2.2bn
Dagmar (Patrick)	26-12-2011	0.04bn	0.05bn	0.2bn
Ulli	3-1-2012	0.2bn	0.2bn	0.9bn
Christian (St Jude)	28-10-2013	1.3bn	2.0bn	3.3bn
Xaver	5-12-2013	0.9bn	1.5bn	3.7bn

Another cause of lower estimates are the remaining gaps in building footprint coverage of OSM (see **Methods**). One of the core issues with OSM is the spatial variability in its completeness^21^. Where urban areas tend to be well-mapped and complete, rural areas are often still lagging behind. Still, several studies have shown the success of OSM over the last few years. Tian *et al*.^22^ show that building count in OSM increased by almost 20 times between 2012 and 2017. Brovelli and Zamboni^23^ found for the region of Lombardy (Italy) a 57% overlay between OSM and an authoritative dataset. However, they also found that around 9% of the buildings in OSM were not in the reference dataset, indicating that OSM could help to fill in the missing gaps in authoritative maps. In our study, we find ~100% building coverage compared to the official statistics in, for instance, The Netherlands, France and Czech Republic (Table S2). Accounting for the current incompleteness in this analysis is not straightforward, as it is nearly impossible to analyze the completeness of the current building coverage for each region within the windstorm paths. It might very well be that coverage is (near) complete for areas where wind speeds are high, such as in urban coastal areas, even if the overall coverage in a country is low. We show an upper bound estimate for the damages in Table 1, by correcting the estimated damages with the OSM coverage ratio in Table S2. This brings some storms more in line with the XWS catalogue reports, while others appear to overestimate the damages. Note that the XWS catalogue shows insured damages, while our method produces overall damages, so an overestimation can be expected. With the continuous improvement of OSM, the estimates are expected to improve over time.

## Discussion and conclusion

This study presents a first high-resolution damage model to estimate the damages to buildings due to extratropical windstorms in Europe. The approach provides flexibility in the derivation by developing the vulnerability curves from building level upwards. The approach is particularly valuable to support insurers’ and academic assessments for post-disaster quick-scans and estimates of potential wind damage towards the future, allowing them to use an open-source and transparent approach. While we demonstrates the methodology on a continental scale, it is not bound by a geographic region, and thus can be applied globally provided that data is available. Moreover, the risk framework applied is similar to those of other hazards, such as flooding^24^ or earthquakes^25^, which means our open source methodology using OSM data is transferable to other hazards.

The damage and risk estimates show that mainly the countries on the western part of Europe are heavily impacted by extra-tropical storms, of which the United Kingdom, Ireland, Germany, France, the Netherlands and Denmark are the most damaged countries. As soon as one moves inland, the damages become substantially lower for most countries. Outliers are Austria and the Czech Republic, which may be due to suboptimal vulnerability curves or exposure values. To calibrate, additional observed data is required to estimate the ‘optimal’ vulnerability curves for these countries. As such, the estimates from this study can be interpreted as a baseline for further developed of an open-source windstorm damage model.

The sensitivity analysis shows that for each country/storm combination, the fragility curves are the most important driver of the results. This does not come as a surprise, as they define when we observe damage at certain wind speeds. The availability of fragility curves is, unfortunately, limited. Fragility curves are often estimated and calibrated using insurance data that is not publicly available. While this damage data could also be collected by governmental agencies, or through scale model experiments in wind tunnels, this would be an expensive enterprise. In contrast, insurance companies are uniquely positioned for fast and cost-effective data collection. Considering the dependency of risk modelling on insurance data, it should be debated whether this information needs to be made more publicly available to improve open access disaster risk reduction efforts.

The still incomplete parts of the OSM database coverage is not consistent throughout Europe, and it can be expected that some results are an underestimation. We account for this incompleteness in Table 1 by showing upper damage estimates. We expect these estimates to improve in the future as the OSM database is constantly evolving and growing. Especially if building stock data is made publicly available by more governments, such as done in The Netherlands and France, OSM data will significantly improve. Furthermore, remote sensing by satellite imagery is improving rapidly with increasing resolution, which can serve as input for OSM data. Even taking the current caveats into account, a comparison with the observed damages from the XWS catalogue and estimates from four vendor models shows that the model performs well. Outliers exist, but overall, the estimates are in the same order of magnitude as previous estimates. Validation shows that the storms that occurred in the 1990s seem to be better estimated compared to the storms that occurred between 2007 and 2010. In conclusion, we suggest that future research should primarily focus on further calibration of the vulnerability curves and exposure values to improve wind damage estimates.

## Methods

The damage estimates are calculated using a conventional risk modelling framework (Fig. S1), where we define risk as a function of hazard – the probability and strength of an event with potential to cause harm; exposure – the value of assets subject to the hazard; and vulnerability – the susceptibility of the asset to hazards of a given severity^26,27^.

### Hazard data

The storm footprints are developed by the UK Met Office and are made publicly available within the WISC project^28^. The footprints are maximum 3-second gust footprints over a 72-hour period and are developed using the ERA Interim and ERA-20C re-analyses. These re-analyses are dynamically downscaled using the UK Met Office Unified Model^29^, covering Western Europe and the North-East Atlantic. The 72-hour period is centered on the time the tracking algorithm identified as the maximum 925 hPa wind speed over land within three degrees of the track center. Each footprint describes the maximum 3-second gust in m/s at each grid point in the downscaled model domain over a 72-hour period. Footprints are available in NetCDF and TIFF format for each storm^28^. Grid points are located on a regular grid in a Cartesian coordinate system, with horizontal grid spacing of 0.04 degrees (approximately a 4.4 km resolution). This provides clear footprints and emphasizes the higher spatial resolution provided by the WISC project compared to the earlier XWS database, as well extending the timeline into the past using ERA-20C. The selection of storms for downscaling used a similar approach to the selection for the XWS catalogue, which involved taking the known ‘insurance events’, plus some strong events exceeding a wind speed threshold along the storm track. See Table S1 for an overview of all wind storms included in this study.

### Exposure data

All building footprint data are extracted from OSM, which has proven to be the most extensive dataset of publicly available building footprints for Europe. Table S2 shows an overview of the number of buildings per country that are included in this analysis. For several countries, such as the Netherlands, Czech Republic and France, OSM data provides us with an almost complete nationwide coverage. For some countries, such as Belgium and Denmark, buildings are missing but coverage is still sufficient for the scales required. Full coverage for a certain region or country is primarily driven by the responsible public authority in a given region or country. If they decide to publish the building database publicly, it is only a matter of time before it will be included into OpenStreetMap. This happened, for instance, in the Netherlands and France. For countries where the building database is not publicly shared, such as the United Kingdom, the OSM database primarily depends on users who geo-reference buildings manually. It should be noted that using present-day exposure estimates to assess the impacts of an event is a common practice in the insurance industry^20^. This may result in an overestimation of the damages due to increased building exposure over time.

To get a sense of the completeness of the OSM building stock, we compare the OSM building count with the reported building stock from the EU building database^30^. This database contains information on dwellings and non-residential buildings. Dwellings are places of residence such as a house, flat or an apartment. Table S2 shows that countries like Austria, the Netherlands, France, Switzerland, and Poland have good coverage, while Spain, Portugal, and the United Kingdom have a low coverage. Besides the previously mentioned argument with regards to the public sharing of data by authorities, another explanation for low(er) coverage in a country compared to the EU building database, is that the OSM database shows building footprints, and the EU building database reports on dwellings, of which many can be in the same building. This means that the actual coverage of building footprints can be expected to be better than reported here. Countries like the Netherlands and Austria seem ‘overcomplete’, which can be explained by a growth in building stock from 2013, and the inclusion of sheds and other similar outhouse building types in the OSM database. Overall, it is important to acknowledge the limitations of the incomplete database, which likely results in underestimation of damages, while also acknowledging its strength as a consistent database for large-scale windstorm analysis. Overall, we consider the OSM dataset as a good starting point that provides building coverage in an open access dataset with coverage on an EU-wide level. Additionally, due to its almost real-time updates, the dataset will only further improve in the future.

As the OSM data does not provide EU-wide coverage of building types, other datasets have to be used to fill this gap. The first step is to identify the potential use of the building. As of now, the best European data set to do so is the Corine Land Cover (CLC) dataset, developed by the European Environmental Agency^31^. This data set distinguishes 45 different land-use classes, varying from high-density residential areas to several different agricultural land-use classes. One benefit of using the CLC dataset is the transparency of its creation. For each land-use class, it is known what percentage of each cell consists of residential, commercial, industrial and various other land uses. This is consistent for the whole of Europe and country-specific. These percentages are used to identify the relative share of each of these potential building uses for every footprint (see Fig S2). By combining the footprint data with CLC data, we can assign use categories to buildings based on their location; i.e. buildings are categorized as low/high density residential, commercial/industrial or agricultural.

The PAGER database^32^ is used to add additional exposure characteristics to the buildings. This global database provides information on the main construction types for buildings in each country. More specifically, the database provides information on the specific use of building types for (i) urban residential, (ii) urban non-residential, (iii) non-urban residential and (iv) non-urban non-residential. The database differentiates between 106 different building types. In practice, it means that we assume that the building type of high-residential urban area in the Netherlands relates to the building types specified as urban residential for the Netherlands in the PAGER database.

### Vulnerability data and damage estimates

Using the building characteristics as a starting point, we can estimate the potential damages. By applying the methods and fragility curves proposed by Feuerstein *et al*.^12^, we use fragility curves for different building construction types as shown schematically in Fig. S2. Linking between the fragility curves proposed by Feurenstein *et al*.^12^ and the damage per building type is done by aggregating the 106 different building types of the PAGER database to the six different building types considered in Feuernstein *et al*.^12^. These building types are (i) weakest outbuildings, (ii) outbuilding, (iii) strong outbuilding, (iv) weak brick structure, (v) strong brick structure and (vi) concrete building. It should be noted that most of the European buildings fall in the last two categories.

To estimate damages, we need to move from fragility curves to vulnerability curves and thus add a monetary value to the potential damages. The first step in moving from the fragility to the vulnerability curves is to use estimated maximum reconstruction costs per building type/construction type. The estimated reconstruction costs are taken from the study performed by Huizinga *et al*.^33^. In their study, they have estimated the maximum reconstruction costs for several building types for each country in the world. From their study, we have taken the values for the European countries considered in this project for (i) residential, (ii) commercial, (iii) industrial and (iv) agriculture. Taking this, and by using GDP levels for each NUTS3 region, we can regionally differentiate the reconstruction costs.

Figure S2 illustrates how the entire damage assessment works in practice. The initial step is a simple spatial overlay between all the datasets. This allows us to extract the relevant values from the land-use data and storm footprints for each individual building. By combining the land-use value (and the corresponding detailed percentages of land-use shares (as illustrated in Fig. S2) with the PAGER building construction types, the damage ratio for a given building can be estimated. To estimate the damage in monetary terms, the damage ratio is multiplied by the reconstruction cost of this building type. Finally, damages are adjusted for the relative level of wealth in a region in comparison with the national level. More specifically, the damages are multiplied by the ratio of regional GDP versus the national GDP.

## Uncertainty and Sensitivity Analysis

By performing a sensitivity analysis (SA), it is possible to identify the effect of each parameter on the model output. Parameters that have a large effect should receive additional attention to cope with the uncertainty they introduce, whereas it is justified to keep parameters that have little effect constant^34^. Since both UA and SA require a large amount of repeated model evaluations, we carry them out in a Monte Carlo modelling framework. Within this study, we follow the approach described by Crosetto *et al*.^35^ and Helton^36^ to investigate the uncertainty and sensitivity related to input parameters. They distinguish the following steps: (1) assigning distributions to input parameters, (2) generating samples of different combinations of input parameters, (3) evaluating the model using the generated combinations of input parameters, and (4) analysing the results for uncertainty and sensitivity.

The SA enables us to explore the variation in model output and to allocate the variation in this output to different input parameters, considering the interaction between these parameters. Using SAlib, a publicly available Python library^37^, we perform a Delta Moment-Independent Measure (DMIM) analysis, as developed by Borgonovo^38^ and Plischke *et al*.^39^. This type of sensitivity analysis can be interpreted as a global sensitivity indicator which looks at the influence of input uncertainty on the entire output distribution without reference to a specific moment of the output (moment independence) and which can be defined also in the presence of correlations among the parameters^38^. For a detailed explanation of the DMIM method and its performance we refer to Borgonovo^38^ and Plischke *et al*.^39^.

The main reason for choosing this specific sensitivity analysis method over the more common methods, such as Sobol, is that it allows for a presence of correlation. In this SA, we specifically want to focus on the influence of using different fragility curves and different ratios of residential/non- residential land-use (Table S6). Only fragility curve 2, 3, 4 (Fig. S2) are included, as manual testing shows that the steepest fragility curve (curve 1) almost always results in damages that are too high, whereas the least steep curves (curve 5 and 6) almost always results in damages that are much lower compared to observed damages.

The parameters listed in Table S6 have a close correlation with each other. The sum of the share of the curves, for instance, should always be 100%. More specifically, the total damage calculated per building is based on a specific share of each of the curves. Each curve represents a specific building type, with a specific relation between wind speed and damage. Unfortunately, there is no publicly available dataset containing the exact building type of each building in Europe and the values included in the PAGER database are countrywide. One can, however, imagine that this is in reality not homogenous over a country. As such, we want to identify the extent to which the damage change if we change the building construction type (e.g. change the fragility curve). As we only have a small set of fragility curves, it is interesting also to allow for a combination of fragility curves. Combining these curves effectively creates a new fragility curve, based on a specific share of each of the existing curves in this new curve. This total share of all curves combined, should be 100%. To illustrate this, Table S7 presents a few examples of potential combinations.

As well as varying the share of fragility curves, we are also interested in identifying to what extent the ratio of residential/commercial versus industry influences the total damage. As shown in Huizinga *et al*. (2017), maximum damage for residential and commercial building types are similar and much higher compared to the maximum damage for industrial building types. The similarity between the residential and commercial reconstruction cost is an outcome of the survey data, used to estimate the reconstruction costs^33^. Because the maximum damages vary between residential/commercial and industrial building types, it is essential to identify the influence of changing this ratio (and thus changing the damages). We are interested in this ratio for both urban and rural areas. In total, we set up a set of 5000 different combinations of parameter values.

It should be noted that these five parameters are not the exhaustive list of all the potential uncertainty in the model. The value of the elements at risk (the reconstruction costs) are a potential source as well. There are, however, two reasons why we focus on the fragility curves. Firstly, according to De Moel *et al*.^40^, the shape of the curve accounts for up to 45 per cent of the total sensitivity in damage modelling outcomes. The value of the elements at risk, on the other hand, only accounted for up to 10 per cent of the total model sensitivity. Secondly, we expect that the fragility curves are more likely to be adopted by the end-users than the potential value of the elements at risk.

### Calibration process

The outcomes of the sensitivity analysis provide an opportunity to calibrate the parameters that are tested. By comparing the 5000 outcomes of the sensitivity analysis with the average damage estimates of four vendor models^20^, we can identify which specific set of parameter settings provides us with the best match. It should be noted, however, that it may well be that the ‘best’ parameter setting for a country to estimate the impacts of one storm may not be the ‘best’ parameter setting for another storm. This is particularly a problem with storm Kyrill, where a lot of local convective activity was observed during the storm. This local activity is not modelled in our storm footprints, but caused the highest damages during storm Kyrill. Hence, the ‘best’ settings for storm Kyrill are most likely quite far off from the other storms. To deal with this issue, we aim to identify the parameter settings that produce the most sensible outcomes for as many storms as possible for a specific country. A one-size-fits-all approach is unfortunately impossible to achieve in such damage modelling frameworks. Tables S3 and S4 in the Supplementary materials show the outcome of the calibration process and the parameter settings used in the results presented in the this paper.

## Supplementary information

Supplementary Information.

## Data Availability

The full code of the model is available through https://wisc.readthedocs.io/en/latest/. All building footprints can be extracted from OpenStreetMap. The date of extraction for this study is July 1, 2018. All hazard data can be obtained through the Copernicus WISC Windstorm Information Service: https://wisc.climate.copernicus.eu/wisc/.
