# Supplementary material for: A high-resolution wind damage model for Europe
Source: Sci Rep. 2020 Apr 22;10:6866. doi: 10.1038/s41598-020-63580-w (PMC7176694; doi:10.1038/s41598-020-63580-w)
Supplement: Supplementary file 1 — Supplementary Information. [file 41598_2020_63580_MOESM1_ESM.docx]

# Supplementary information

A high-resolution wind damage model for Europe

Koks & Haer (2019)

**Table S1 | Overview of all storms considered in this study**

| **Date** | **Storm Name** | **Date** | **Storm Name** |
| --- | --- | --- | --- |
| 1981110215 | Nov-81 | 1996110518 | Nov-96 |
| 1983011812 | Jan-83 | 1997032800 | Mar-97 |
| 1983020118 | Feb-83 | 1997122418 | Yuma |
| 1984011303 | 13-Jan-84 | 1998010412 | Fanny |
| 1984011421 | 14-Jan-84 | 1998102800 | Xylia |
| 1984112321 | Nov-84 | 1998122618 | Stephen |
| 1986012006 | Jan-86 | 1999120318 | Anatol |
| 1986032500 | Mar-86 | 1999122606 | Lothar |
| 1986102200 | Oct-86 | 1999122718 | Martin |
| 1987101606 | 1987J | 2000103003 | Oratia |
| 1988020821 | Feb-88 | 2002012715 | Jan-02 |
| 1988112918 | Nov-88 | 2002102715 | Jeanette |
| 1990012515 | Daria | 2005010712 | Erwin |
| 1990020309 | Herta | 2005011121 | Gero |
| 1990020800 | 08-Feb-90 | 2007011815 | Kyrill |
| 1990021121 | 11-Feb-90 | 2008020112 | Resi |
| 1990022618 | Vivian | 2008030200 | Emma |
| 1990022818 | Wiebke | 2009012406 | Klaus |
| 1991010818 | 08-Jan-91 | 2009021103 | Feb-09 |
| 1992112506 | Nov-92 | 2010022712 | Xynthia |
| 1993011315 | 13-Jan-93 | 2011121600 | Dec-11 |
| 1993012303 | 23-Jan-93 | 2011122603 | Dagmar |
| 1993120818 | Dec-93 | 2011122812 | Patrick |
| 1994012806 | Lore | 2012010300 | Ulli |
| 1995011803 | Jan95_2 | 2013102815 | Christian |
| 1996020712 | Feb-96 | 2013120515 | Xaver |
| 1996102718 | Oct-96 |  |  |

**Table S2 | Overview of OSM building footprint coverage per country.** Comparison of OSM building footprints with reported building stock from the EU buildings database (EU-BD). The values are based on 2013 which is for many countries the latest reported year of building stock. The reported building stock combines dwellings and non-residential buildings.

| **Country** | **OSM**  **Building footprints** | **EU-BD (2013)**  **Dwellings + non-residential buildings** | **OSM / EU-BD** |
| --- | --- | --- | --- |
| AT | 3718784 | 3573439 ^1^ | 1.11 |
| BE | 2826675 | 6057240 | 0.47 |
| CH | 2279825 | 1859893 ^2^ | 1.23 |
| CZ | 4568244 | 5065583 ^1^ | 0.90 |
| DE | 28793010 | 43981240 | 0.65 |
| DK | 2009119 | 3124680 | 0.64 |
| EE | 682956 | 681220 | 1.00 |
| ES | 2196458 | 28079660 | 0.07 |
| FI | 1496195 | 3132670 | 0.48 |
| FR | 46965615 | 34996650 | 1.34 |
| GB | 6359272 | 29420210 | 0.21 |
| IE | 588847 | 1769570 | 0.33 |
| IT | 11455829 | 33845900 | 0.34 |
| LT | 923877 | 1377670 | 0.67 |
| LU | 113257 | 231230 | 0.49 |
| LV | 437803 | 1114394 ^1^ | 0.42 |
| NL | 10221164 | 8298650 | 1.23 |
| NO | 742508 | 2205191 ^3^ | 0.34 |
| PL | 10620612 | 14734280 | 0.72 |
| PT | 805327 | 6544550 | 0.12 |
| SE | 1986158 | 5423710 | 0.37 |

1 Building stock missing from EU-DB. Building stock (2001) obtained from Eurostat (2018).

2 Actual building count obtained from the Federal Statistical Office Switzerland (2018)

3 Building stock (2001) obtained from Statistics Norway (2011). Missing values for non-residential buildings. Non-residential building count is estimated based on the average share non-residential / dwellings of other countries.

**Table S3 | Ratios of vulnerability curves which provided outcomes that were in the similar order of magnitude as estimated losses through the vendor models**

| Country | c2 | c3 | c4 |
| --- | --- | --- | --- |
| Austria | 5% | 0% | 95% |
| Belgium | 0% | 45% | 55% |
| Denmark | 0% | 20% | 80% |
| France | 0% | 15% | 85% |
| Germany | 5% | 50% | 45% |
| Ireland | 30% | 70% | 0% |
| Luxembourg | 50% | 50% | 0% |
| Netherlands | 0% | 45% | 55% |
| Norway | 0% | 100% | 0% |
| Finland | 5% | 15% | 80% |
| Poland | 5% | 15% | 80% |
| Sweden | 0% | 10% | 90% |
| Italy | 10% | 90% | 0% |
| Spain | 15% | 85% | 0% |
| Portugal | 15% | 85% | 0% |
| United Kingdom | 5% | 15% | 80% |

**Table S4 | Ratios of Residential & Commercial vs industrial for both urban and non-urban land-uses**

|  | Urban land-use | | Rural land-use | |
| --- | --- | --- | --- | --- |
| *Country* | Urban residential & commercial | Urban non-residential | Non-urban residential & commercial | Non-urban non-residential |
| Austria | 80 | 20 | 60 | 40 |
| Belgium | 70 | 30 | 60 | 40 |
| Denmark | 80 | 20 | 60 | 40 |
| France | 80 | 20 | 60 | 40 |
| Germany | 70 | 30 | 60 | 40 |
| Ireland | 70 | 30 | 60 | 40 |
| Luxembourg | 80 | 20 | 60 | 40 |
| Netherlands | 80 | 20 | 60 | 40 |
| Norway | 80 | 20 | 60 | 40 |
| Finland | 50 | 50 | 60 | 40 |
| Poland | 70 | 30 | 60 | 40 |
| Sweden | 70 | 30 | 60 | 40 |
| Italy | 80 | 20 | 60 | 40 |
| Spain | 80 | 20 | 60 | 40 |
| Portugal | 80 | 20 | 60 | 40 |
| United Kingdom | 50 | 50 | 60 | 40 |

Table S5 | Overview of average losses per storm, per country, as estimated by four vendor models (IF, CoreLogic, RMS and Air). Values based on the study of Waisman (2015).

|  | Anatol | Daria | Klaus | Kyrill | Lothar |
| --- | --- | --- | --- | --- | --- |
|  | 3-12-1999 | 25-1-1990 | 24-1-2009 | 18-1-2007 | 26-12-1999 |
| Austria | 1 | 5 | 6 | 317 | 64 |
| Belgium | 21 | 874 | 4 | 226 | 18 |
| Denmark | 2553 | 167 | 3 | 22 | 4 |
| France | 5 | 704 | 1440 | 208 | 7353 |
| Germany | 227 | 1537 | 42 | 2310 | 1082 |
| Ireland | 20 | 108 | 2 | 143 | 2 |
| Luxembourg | 1 | 22 | 0 | 8 | 10 |
| Netherlands | 47 | 1673 | 4 | 383 | 11 |
| Norway | 10 | 5 | 2 | 4 | 10 |
| Sweden | 240 | 49 | 0 | 1 | 1 |
| Switzerland | 0 | 21 | 43 | 39 | 1020 |
| United Kingdom | 208 | 5978 | 1 | 523 | 55 |
| Total | 3331 | 11142 | 1546 | 4184 | 9630 |

**Table S6 | Parameters used in the sensitivity analysis**

| Parameter | Description | Range |
| --- | --- | --- |
| c2 | Curve 2 - Outbuildings | 0-100 |
| c3 | Curve 3 – Strong Outbuildings | 0-100 |
| c4 | Curve 4 – Weak brick structures | 0-100 |
| lu1 | Ratio residential/non-residential in urban areas | 0-50 |
| lu2 | Ratio residential/non-residential in rural areas | 0-50 |

| **Table S7 \| Three examples of parameter value combinations (in percentages)** | | | | |
| --- | --- | --- | --- | --- |
| **c2**  **Curve 2** | **c3**  **Curve 3** | **c4**  **Curve 4** | **lu1**  **Ratio urban** | **lu2**  **Ratio rural** |
| 50 | 30 | 20 | 40 | 30 |
| 10 | 70 | 20 | 10 | 50 |
| 60 | 10 | 30 | 30 | 20 |


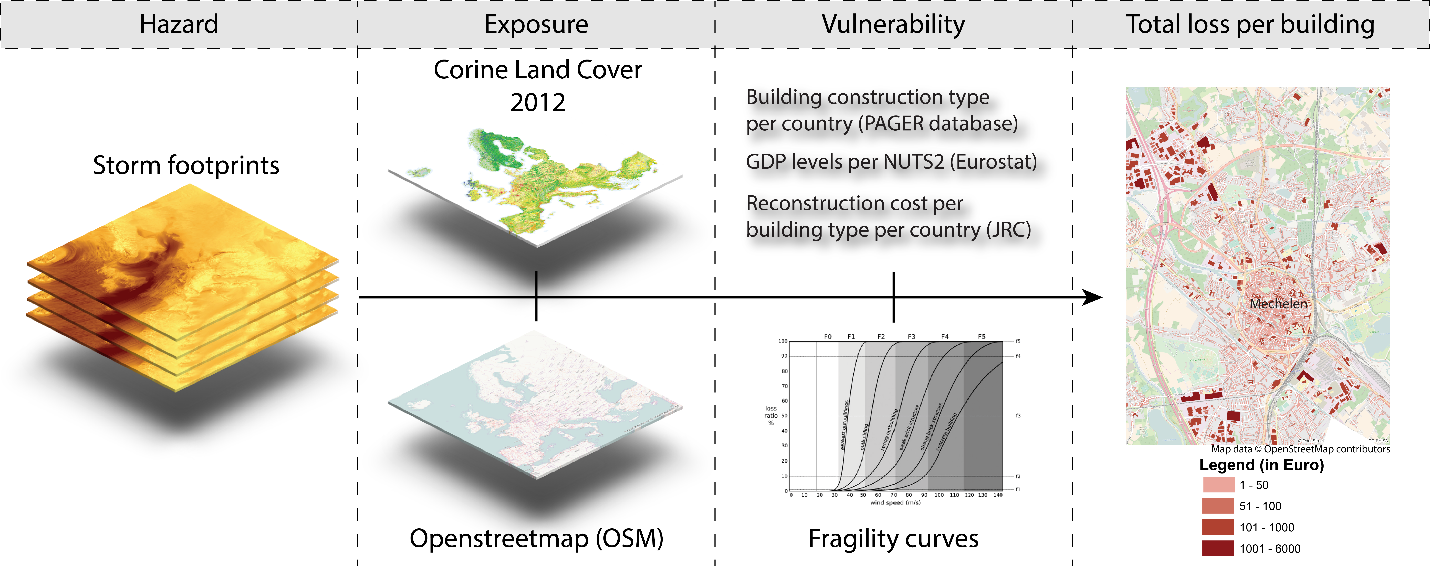


**Fig S1 | Overview of the various steps for the loss estimation.**


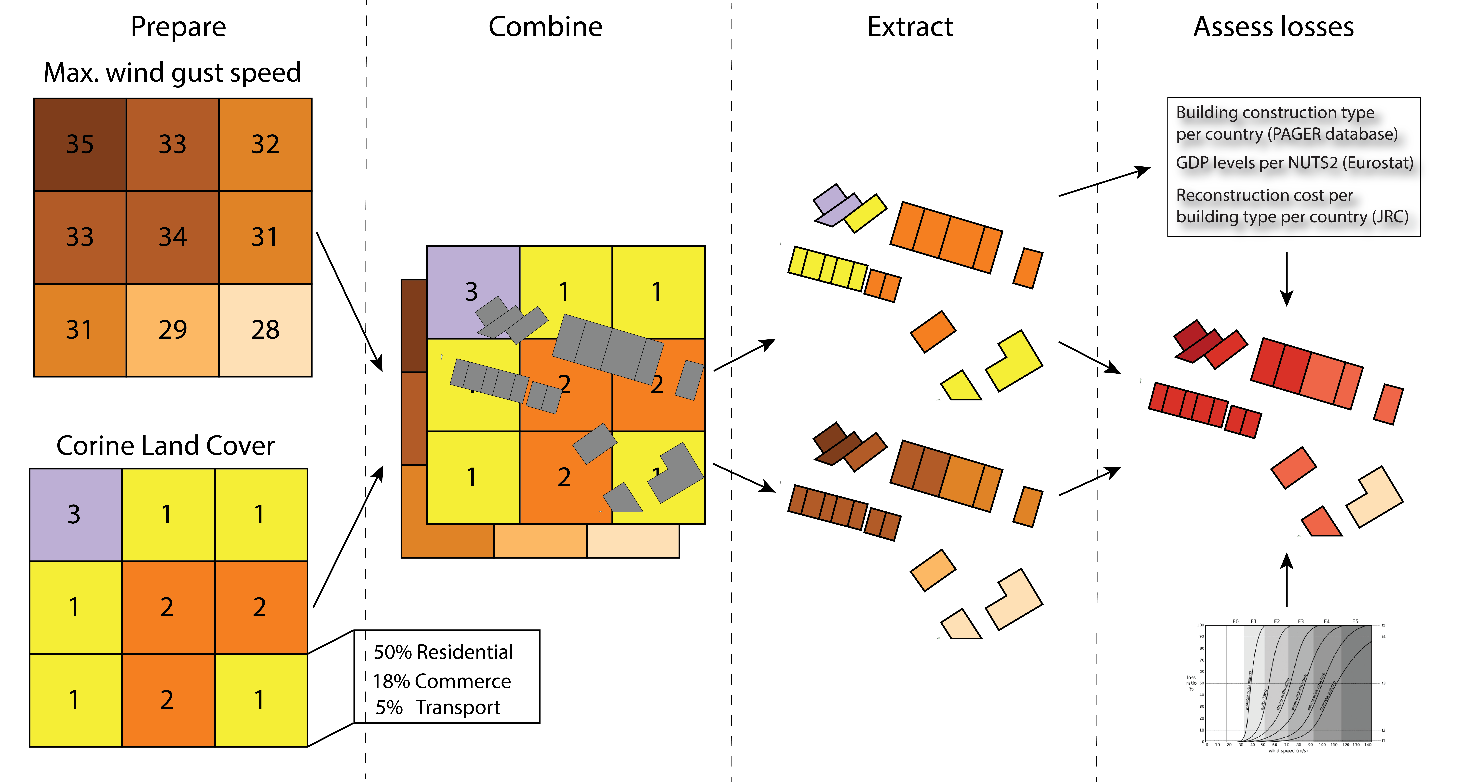


**Fig S2 | Practical overview of the damage calculation**


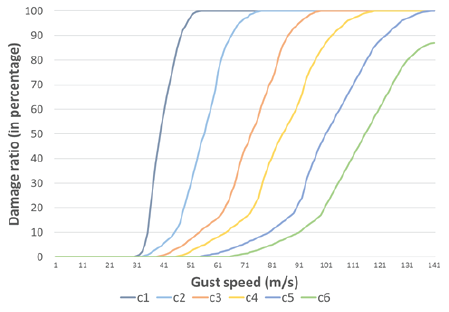


**Fig S3 | The six fragility curves considered in this study. Based on Feuerstein et al. (2011)**

**
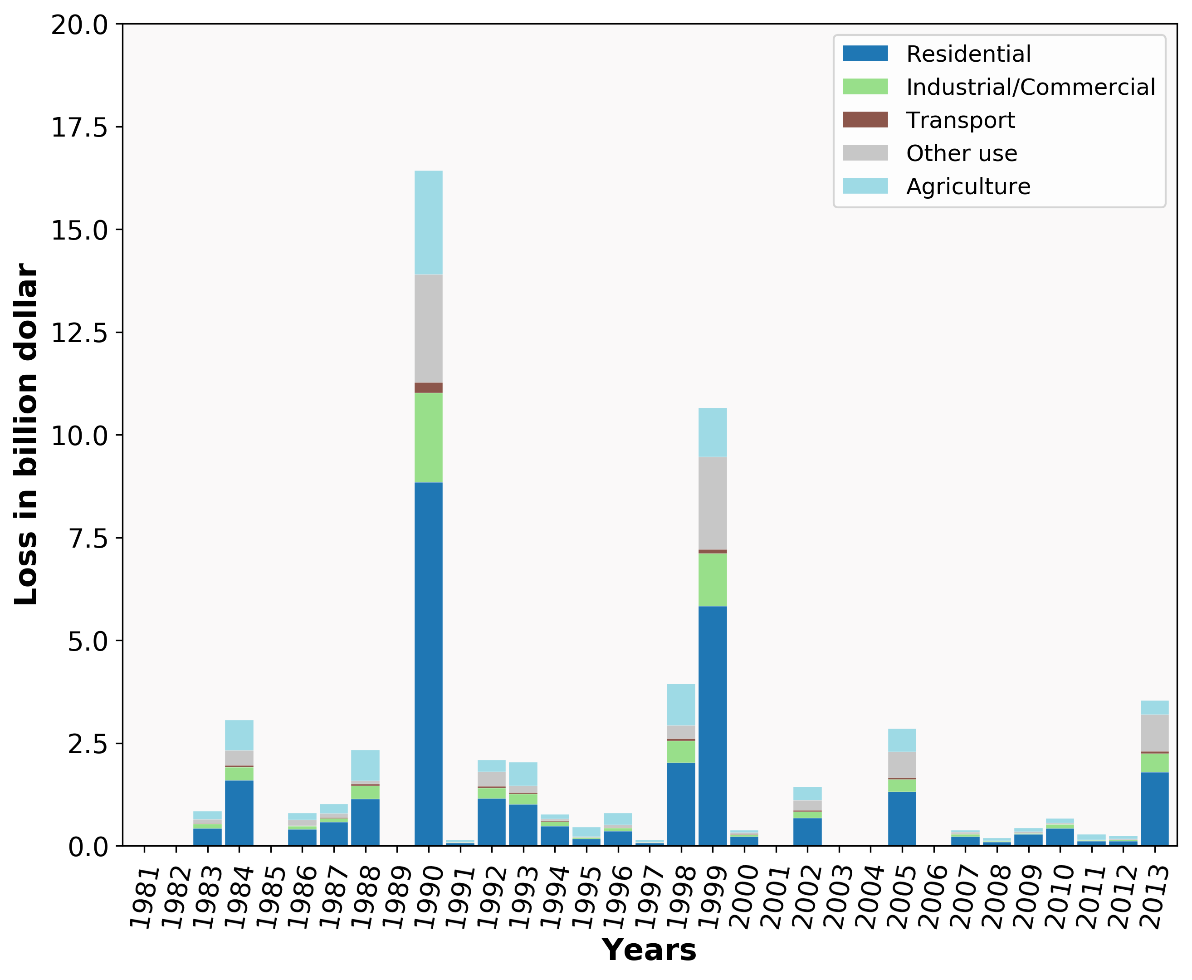
**

**Fig S4 | Historical damages per sector per year**
